# Course and severity of bipolar disorder determining TGF-beta

**DOI:** 10.1192/j.eurpsy.2025.786

**Published:** 2025-08-26

**Authors:** S. Dodic, M. Velimirovic Bogosavljevic, V. Jurisic, M. Nesic, M. Gostiljac, M. Puric, I. Minic, B. Velickovic, B. Dunjic-Kostic, M. Ivkovic, M. Pantovic-Stefanovic

**Affiliations:** 1 Clinic for Psychiatry, University Clinical Centre of Serbia; 2 School of Medicine, University of Belgrade; 3 Institute of Clinical and Medical Biochemistry, Belgrade; 4 Faculty of Medical Sciences, University of Kragujevac, Kragujevac; 5 Institute of Mental Health, Belgrade, Serbia

## Abstract

**Introduction:**

Bipolar Disorder (BD) is a severe and chronic psychiatric condition, with complicated course and substantially reduce psychosocial functioning. Alterations in immune response are considered to be one of the major factors underlying the etiopathogenesis of BD, causing alterations in neurotransmitter systems, neuroendocrine systems, neurotrophic factors, and producing oxidative stress. Inflammatory cytokines have potential value for the clinical diagnosis and prognosis of BD.

**Objectives:**

The primary aim of the study was to assess the effect of disease course and clinical-characteristics on alterations of transforming growth factor-beta (TGF-beta), in BD.

**Methods:**

This cross-sectional study included 82 patients with BD in remission. Multivariate linear regression with TGF-beta value as an outcome, and duration of illness, number of hospitalizations, residual symptoms estimated as Brief Psychiatric Rating Scale (BPRS) score, gender, and age, was used to produce the model.

**Results:**

The explored linear regression model was significant, explaining 39.4% of the variance (p<0.001). Higher TGF-beta was predicted by less previous hospitalizations (p<0.001) and lower BPRS score (0.034), while longer duration of illness was almost significant predictor (p=0.054). Age and gender showed no predictive effect in the model.

**Conclusions:**

The study points out to the better course of BD characterized by less episodes and less residual symptoms in determining the TGF-beta levels, potentially creating a more favorable immunological state. The importance of neuroprotective and neurodegenerative balance of immune mediators, their interplay and relation to chronicity and severity of BD should be further explored.

**Disclosure of Interest:**

None Declared

